# Empyema of the Accessory Sinuses of the Nose*Read before the Southern Section of the American Otological, Rhinological and Laryngological Association, Atlanta, March 28th.

**Published:** 1898-05

**Authors:** Frank M. Hanger

**Affiliations:** Oculist and Aurist to the State Deaf, Dumb and Blind Institution, Staunton, Va.


ATLANTA

Medical and Surgical Journal.

Vol. XV. MAY, 1898. No. 3.

L. B. GRANDY, M.D., DUNBAR ROY, M.D., } EDITORS. M. B. HUTCHINS, M.D., BUSINESS MANAGER.

### ORIGINAL COMMUNICATIONS.

### EMPYEMA OF THE ACCESSORY SINUSES OF THE NOSE.*

By FRANK M. HANGER, M.D., Staunton, Va.,

Oculist and Aurist to the State Deaf, Dumb and Blind Institution, Staunton, Va.

Much has been written concerning these affections, and we feel as if the field has already been exhausted. But after reviewing the literature on the subject and noting the diversity of opinion expressed and the contradictory conclusions that have been formed by rhinologists of equal prominence, we are at a loss to know whom to follow and what creed to adopt. Owing to this fact, we believe we should be guided more by our own experience and observation than by the dogmas of any man, however eminent; and we shall make effort to observe this principle in our remarks, trusting our views will at least merit the approval of some, while at the same time we incur the criticism of many.

Before presenting our subject in detail, there are a few general remarks common to all the accessory sinuses of the nose.

*Read before the Southern Section of the American Otological, Rhinological and Laryngological Association, Atlanta, March 28th.

They all communicate with the nasal fossæ by small openings, are alike filled with air, lined with the same mucous membrane, and therefore liable to be invaded by any of the inflammations that may affect these passages.

In view of these facts, we think we can safely claim that by far the largest percentage of empyemas is caused by influenza and coryza. As a rule the sinuses escape these inflammations, but at times they are undoubtedly involved. The inflamed cavity becomes filled with mucus which, finding no outlet on account of the swollen and occluded condition of the natural opening, undergoes decomposition, and the mucous lining becomes reinfected and converted into a pyogenic membrane. Now unless the natural opening is soon restored, the periosteum may become diseased by contiguity of structure, or its function interfered with by the pressure of the accumulating fluid; thus, necrosis of the bone is the inevitable result.

It is thus possible to explain the presence of granulations, polypi, and myxomatous degeneration, so frequently associated with purulent affections of these cavities. However, empyema may be caused primarily by lesions in the walls of the sinuses themselves, such as trauma, syphilis, tuberculosis, metastasis of pyæmia, and various depressing diseases which are apt to be accompanied or followed by suppuration. Atrophic rhinitis is a factor which must not be forgotten.

In addition to the above causes, empyema of the maxillary sinus alone enjoys the distinction of dental origin.

These affections are distinctively of adult life. Seldom do we meet with cases under twenty years of age, and most of them range between twenty-five and sixty. The reason of this peculiarity, we think, is apparent: As we grow old, repeated colds and influenzas generally leave the mucous membrane of the nose more thickened and congested, resolution less complete with each attack, and the openings of the sinuses less patulous, so that accumulated fluids find more difficulty of egress and consequently remain in the cavities longer than in youth, and thus the chance of infection is increased.

Then, again, as we grow old the carious condition of our teeth is responsible for many cases of maxillary sinusitis. We are seldom called on to treat these affectious in the acute stage. As a rule, *vis medicatrix naturæ* is sufficient, and as the rhinitis which produces them subsides, the cavities are emptied and a spontaneous cure results. We have on more than one occasion successfully emptied the antrum of Highmore of a muco-purulent collection, the result of acute coryza, by alternately lowering the head and holding it to the opposite side, at the same time blowing the nose.

After the empyemas have lasted some weeks or months, the discharge becomes more purulent and creamy in appearance, at times caseous and mealy, with a marked fetid odor; and it is usually this odor which drives our patients to seek relief, after they have become a nuisance to themselves and their friends.

One or more of the sinuses may be affected at the same time, on the same side, or may be bilateral. It is possible for one sinus to be infected by pus arising from another, and on several occasions we have witnessed violent purulent otitis media as a result of pus infection from the maxillary antrum.

Furthermore, empyema of those cavities which lie at the base of the brain are at times responsible for meningitis and cerebral abscess.

Successful diagnosis and treatment is what concerns us most, and we will now consider the best means of reaching this end, in each one of the accessory sinuses, and will beg your indulgence while we present, first—

### EMPYEMA OF THE ANTRUM OF HIGHMORE.

This cavity, as well as the sphenoidal sinus, is specially prone to become filled with accumulated fluids because its natural opening is near the top.

Our patient comes to us complaining of a fetid, pus-like discharge, usually from one nostril, which has lasted for some weeks or perhaps years, dating from an attack of influenza or cold. In the morning his throat is filled with the discharge, and at intervals during the day, on stooping over, it runs out of the nose. He may or may not complain of a sense of fullness under the corresponding eye, and at times pain and tenderness along the malar bone and extending back to the temple. This pain and sense of distension is very pronounced in the acute stage, but as the disease becomes chronic, our patients are remarkably free from discomfort and reflex pains. On examination we usually find pus in the upper middle part of the middle meatus and slowly trickling down over the inferior turbinated bone. The fact that the pus is not found higher up in the nasal passage points to the non-involvement of the sphenoidal sinus and the ethmoidal cells.

Now spray the nose with some alkaline wash and follow with a 4 per cent. solution of cocain. This makes manipulation painless and opens up the nasal passages to better inspection. With a piece of cotton on the end of a probe, mop all pus from the site of the hiatus semilunaris and wait. It will not be long before a drop of pus will ooze from the opening. Then direct the patient to hang the head down and shake it back and forth, and from side to side. On looking into the nose again, you will usually find the passage filled with a creamy pus in such quantity as to exclude the possibility of its origin from any of the other sinuses.

At times the character of the pus varies. It may be sero-purulent, muco-purulent, caseous, mealy, or muco-gelatinous. The last named is seldom dislodged from the cavity without irrigation; it comes away *en masse* and is of such consistency as not to be even broken up with a spray of water.

The teeth should now be examined to see if any are carious or offending sufficiently to be responsible. If there is still any room for doubt as to a diagnosis, because of the failure to get pus, the antrum should, if possible, be irrigated after first locating and testing the size of the ostium with a probe-pointed sound. If this is practicable, we are able to evacuate the pus at once and the case is made clear. Should we fail, we can use the electric trans-illumination test after the method of Heryng or Voltolini. Should there be pus in the antrum, we should expect the corresponding cheek to be less illuminated than its fellow and a dark crescent appear just below the lower eyelid, and also the subjective luminous perception be less in the corresponding eye.

This test is merely corroborative, and is often misleading or negative. Should we have a tumor in the antrum, we get all of these signs, only intensified. As the extraction of pus is the only infallible sign of empyema, we much prefer exploration with the trocar through the middle or inferior meatus, or even the canine fossa. The inferior meatus is safer than the middle, as there is less likelihood of wounding the orbit.

We have been able to cure more than half of our cases, by irrigation through the natural opening, and give this method the preference, not only in every case of antral empyema, but in all the other sinuses. If you fail to cure by this method, you will seldom succeed by irrigating through an artificial opening of whatever selection. Catheterizing the hiatus semilunaris is by no means an easy task; still, it is not only possible, but a practicable procedure, and is more easily accomplished than a like result in any of the other natural openings.

Telling how these catheterizations are done, and putting them into effect, are two entirely different things. It requires a delicate hand and the most skilful manipulation, and, even then, we fail in many cases. After we have learned the contour of the nose, in each special case, it becomes comparatively easy.

The canula for irrigating the antrum should be of sufficient length and bent, at a right angle, one-third of an inch from the extremity, and connected with an india-rubber bulb-syringe that will hold about an ounce of fluid. The canula is introduced into the nasal passage with the beak upward, and then rotated outward into the ostium.

When in position, and the syringe already filled with the irrigating fluid, direct the patient to inhale deeply and hold the breath, at the same time bend the head forward. When the syringe is nearly emptied, direct the patient to exhale through the nose. This simple precaution will prevent the terrible strangling we have often witnessed. For irrigation, there is nothing better than a two or three per cent. solution of boric acid. In obstinate cases, a one per cent. solution of nitrate of silver, or peroxide of hydrogen, pure or diluted, may be used with advantage, after first cleansing the cavity with the boric acid solution, and following with a second irrigation of the same. Treatment, by this method, usually lasts from one to eight weeks, daily irrigations being used. The time required to effect a cure, bears no regular proportion to the period the empyema has existed. We have seen a case of thirty-two years' standing cured in two weeks, and one of two weeks' standing require as many months of treatment.

Should the hiatus semilunaris not be accessible or patulous enough for irrigation, our next preference is an opening made through the alveolar process, at a point where a bicuspid or a first or second molar tooth has already been extracted, or should one of said teeth be so carious as to require extraction, or be the probable cause of the empyema. .Under no circumstances should a sound tooth be sacrificed. For making the opening, a hand drill and mastoid curette are all that is needed.

The point of the drill should be directed upward and a little inward. A drainage-tube has little advantage over a plug, and either can be used with good results. The former permits irrigation while *in situ,* but little drainage takes place from it after a few days, and has the disadvantage of permitting extraneous matter entering the cavity, unless provided with a suitable obturator during meals. The tube or plug should be removed as soon as possible after discharge has ceased, but the irrigation should be continued as long as the wound remains open.

Some object to the alveolar opening because it is so long closing. We have never noticed this difficulty in the least, and it usually has a marked tendency to close before we are ready. The patient himself can perform the irrigation much better than through an opening made in the inferior or middle meatus.

Our next preference is an opening through the inferior meatus, made with the drill and curette. This can also be made with the electric cautery.

This method has the advantage of permitting no pus dripping into the patient's mouth or particles of food entering the cavity. Besides, the patient can be taught how to irrigate. It has the disadvantage of being more difficult to perform, and at times impossible, on account of the interference of the inferior turbinated, and the excessive thickness of the bone. There is little danger of wounding the orbit if due care is used.

We have, on two occasions, enlarged the natural opening, in the middle meatus, in an anterior direction, with the electric drill, observing the precaution of pressing the drill downward and outward for fear of entering the obit. Should irrigation fail to cure in two months, and the discharge remain purulent, with a caseous or mealy tendency, an opening should be made in the canine fossa, large enough to permit free inspection and manipulation. Should we discover granulations, polypi, or necrosed bone, the cavity should be thoroughly curetted, irrigated and packed with iodoform gauze, and changed each day until the pyogenic character of the membrane has been destroyed.

We are opposed to the indiscriminate use of the curette, and think it should be reserved for special cases, and thus prevent, as far as possible, the formation of cicatricial tissue.

In most cases the medicated gauze will effect as good result and leave the mucosa in a natural condition. A soft rubber drainage-tube should be introduced and daily irrigations practiced.

For our opening we prefer the canine fossa to the lower border of the molar ridge at the root of the first molar tooth. The bone is thinner in the canine fossa and it is a better site for curetting and reaching all parts of the antrum, although not quite as dependent. Both are equally inaccessible for operation and after-treatment.

### EMPYEMA OF THE FRONTAL SINUS.

Diagnosis in these cases is not always easy, and is often mistaken for, and associated with, purulent troubles of the anterior ethmoidal cells; for, by means of the infundibulum, the middle meatus communicates with the cells, and, through them, with the frontal sinus.

Our patient complains of purulent discharge from the nostril, and the flow is not increased on stooping over, but a sense of frontal fullness and interorbital pain is generally manifest and increased by this posture. If the infundibulum is patulous, in all probability we will observe no external swelling at the inner angle of the orbit and over the superciliary ridges.

Should the natural opening close, or the pus flow be too great to escape, this will occur and the pus will point, usually, toward the orbit at its inner angle. On looking into the nose we will find the anterior part of the middle meatus bathed in pus, coming from the region of the infundibulum and too anterior for the ostium maxillare. If we are not now able to detect dead bone with a probe, we are certain the ethmoid bone is involved, but the frontal sinus may or may not be; and, in some cases, it is impossible to make a differential diagnosis without invading the sinus from the front. If, however, we find no dead bone in the ethmoid, and can succeed in irrigating through the infundibulum and get pus, and the pus flow ceases for half an hour or more, we are certain we are dealing with a frontal sinusitis alone. If the pus flow begins almost immediately, after a successful irrigation, there is ethmoidal complication. If we have a nasal flow of pus and external swelling of the walls of the sinus, there is no longer any room for doubt as to the diagnosis. This trouble may be mistaken for orbital abscess or phlegmonous abscess of the lachrymal sac. In the former ptosis is usually in proportion to the swelling of the lids, while in frontal empyma the ptosis is marked at first and out of proportion to the swelling.

In lachrymal abscess the swelling is too low, and pus points below the tendon of the orbicular muscle. We should always attempt irrigation through the infundibulum, using preferably a boric acid solution. The canula should be bent at a right angle about one-third of an inch from the end, and care should be used not to wound the mucous membrane in its introduction. It should be directed forward and upward and its beak rotated a little outward as it becomes engaged in the narrow portion of the infundibulum. Generally it is best to first remove the anterior extremity of the middle turbinated, in order to facilitate the operation. Should we fail to catheterize and external perforation threaten, we should open the sinus from the front, preserving the periosteum either with the trephine or gouge and mallet, and a free communication established with the nose, using a probe as a guide.

If necessary the cavity should be carefully curetted and packed with gauze until it is deemed safe to close the wound. A drainage-tube should be kept in the nasal opening all the while. As soon as the wound is closed irrigation should be begun through the nose, which is now easily accomplished.

Zaufal recommends the daily evacuation of the pus, with the air douche, through a fine india-rubber tube, after laying bare the entrance of the infundibulum.

Fuchs has dissected out the entire mucous membrane of the sinus, with its anterior bony wall, with good result. Some rhinologists leave the cases to nature, and when pus points on the inner orbital wall, evacuate it there. The possible fistula and the resulting contraction and deformity of the eyelid condemn this let-alone procedure.

### EMPYEMA OF THE ETHMOIDAL CELLS.

Some of the remarks made on the differential diagnosis of frontal empyema from involvement of the anterior ethmoidal cells are applicable here. Pus arising from the posterior ethmoidal cells may be confounded with sphenoidal empyema and is often associated with it. This is specially true when a natural communication exists between these cells and the sphenoidal sinus, as is sometimes the case.

The natural opening into the posterior cells is found in the superior meatus at the front part of the upper wall, and pus proceeding from that point has too high an origin to be mistaken for that which flows from the maxillary ostium.

If we are fortunate enough to irrigate the sphenoidal sinus, which is generally impossible, it will greatly assist in reaching a correct diagnosis, for if this cavity alone be involved, the pus flow will cease for a while, whereas if the ethmoidal cells be affected, the pus flow will reappear almost at once. As an almost invariable rule the probe reveals necrosed bone. This necrosis may be primary in some cases, the result of syphilis, tuberculosis, etc.; but we cling to the opinion that it is, as a rule, secondary and produced by suppurative intra-cellular pressure and faulty drainage.

Owing to the labyrinthine and extensive structure of the ethmoidal cells, it is a physical impossibility to effect their proper irrigation. Frequently associated with ethmoidal empyema are polypi and myxomatous degeneration of the middle turbinated bone. This bone is found enlarged, firmly wedged in between the septum and outer wall of the nose and bathed in a creamy pus. It is this condition which is frequently responsible for asthmatic complications and other reflex neuroses. Our patients complain of the fetid discharge accumulating in the throat during sleep, loss of memory, and deep frontal and interorbital pains which are increased on lowering the head.

There is no disease of the nose, save atrophic rhinitis, which is so intractable to treatment. This is not to be wondered at when we consider the anatomical structure of the bone. There is nothing so essential to the relief and cure of these cases as *drainage*, and this can only be accomplished by thoroughly opening up the cells with a sharp curette, or better perhaps, the drill. All dead bone should be carefully scraped. Care should be taken not to wound the orbit or enter the brain. Until the cells are opened up irrigation is useless. We have never seen a hemorrhage sufficient to justify a tampon, and oppose it because it interferes with drainage and might dam up pus and cause mischief. Polypi or a myxomatous middle turbinated should be removed at a prior operation.

Woakes is a strong advocate of the use of chromic acid applied with a fenestrated platinum probe, wherever dead bone is discovered. The method is too slow, does not reach the site of the trouble, and in our hands has produced no good result. After the cells have been once opened, daily irrigations, with an occasional use of the curette, will do much toward effecting a cure. However, we must confess our failures in this malady far exceed our successes.

### EMPYEMA OF THE SPHENOIDAL SINUS.

The size and extent of the sphenoidal sinuses vary, and at times communicate with the posterior ethmoidal cells in front and extend into the basilar process of the occipital bone as far as the foramen magnum. Empyema of these cavities, we believe, is less often diagnosed than the same trouble in any of the other accessory sinuses. It is most frequently mistaken for purulent affection of the posterior ethmoidal cells, and the remarks on that subject apply here. The natural opening of the sphenoidal cavity lies in the extreme upper back part of the nasal fossa, near the septum and behind the posterior extremity of the superior turbinated bone. It is extremely difficult to catheterize. Before this can be successfully accomplished it is generally necessary to remove the posterior end of the middle turbinated body, which greatly obstructs manipulation. A long straight canula is used. We are of the opinion that primary empyema, without necrosis of the anterior face of the sphenoidal body, is seldom diagnosed and treated through the ostium. As a rule our attention is directed to fungous granulations in this region, and on investigation necrosis is discovered near the natural opening. Our patients commonly complain of little else than post nasal dripping of pus, which accumulates in the throat during the night, and a deep nasal or post-nasal pain. Opening the cavity and irrigation is the proper treatment. The only two cases we have met with were discoverd after removing the middle turbinated bone, for myxomatous degeneration, in the treatment of ethmoiditis. The anterior face of the sphenoidal body was carious in both and fungi marked the site of the diseased bone. With a strong, sharp curette the necrosed bone was scraped and an opening made into the sinus, in both cases. Irrigation was then daily practiced, which effected a cure in one case, after several months of constant treatment. The other passed from our hands before a cure resulted, though somewhat improved.

